# Hemoglobin SE Disease Presenting as a High-Altitude Massive Splenic Infarction Complicated by Hemorrhagic Conversion and Splenectomy

**DOI:** 10.7759/cureus.10321

**Published:** 2020-09-08

**Authors:** Andrew Pajak, Jonathan C Li, Alice Liu, Shaina Nazare, Bruce Smith

**Affiliations:** 1 Internal Medicine, Christiana Care Health System, Newark, USA; 2 Internal Medicine, Sidney Kimmel Jefferson Medical College, Philadelphia, USA

**Keywords:** hemoglobin se disease, vaso-occlusive crisis, splenic infarcts, hemorrhagic conversion, sickle cell trait, hemolytic anemia, sickle cell disease, anticoagulation

## Abstract

Hemoglobin SE (HbSE) disease is a hemoglobinopathy resulting from the combination of hemoglobin S (HbS) and hemoglobin E (HbE) genotypes. It may present as a vaso-occlusive crisis (VOC) in the setting of an acute stressor. Herein, we present a case of undiagnosed HbSE disease presenting as a massive splenic infarct in the setting of high-altitude exposure. A 55-year-old female of South Asian descent presented with acute left upper quadrant abdominal pain after hiking in the Swiss Alps four days previously. Laboratory testing revealed that she had hemolytic anemia, and computed tomography (CT) imaging showed a greater than 50% splenic infarction. After the initiation of anticoagulation, she experienced a hemorrhagic conversion of the initial splenic infarct resulting in acute hemodynamic decompensation. She initially underwent vascular intervention with arterial plugging, coiling, and embolization but ultimately required a splenectomy and partial colectomy upon developing a large splenic hematoma. Hemoglobin electrophoresis was consistent with hemoglobin SE disease. Hemoglobin variants, especially combined heterozygosity, are rare and have the potential to present as a vaso-occlusive crisis in the setting of acute chemical and physiological stresses. Only 43 cases of hemoglobin SE disease have been previously reported and one other occurrence in the setting of high altitude. Conservative management is recommended when a diagnosis of sickle cell trait (SCT) is definite, in comparison with cardioembolic phenomena, in which antiplatelet and anticoagulant therapy should be initiated. Hemoglobin SE disease is a rare heterozygous hemoglobinopathy resulting from the combination of hemoglobin variants geographically separated by thousands of miles. Currently, there are no strict guidelines supporting anticoagulation for the management of VOC in hemoglobinopathies. Splenic infarct in HbSE disease should be managed similarly to SCT/sickle cell disease (SCD) with fluids and analgesia, and anticoagulation should be limited to confirmed thromboembolic events and with the insight of an anticoagulant specialist.

## Introduction

Hemoglobin SE (HbSE) disease is a hemoglobinopathy resulting from the combination of hemoglobin S (HbS) and hemoglobin E (HbE) genotypes. This combination is extremely rare, with only 43 reported cases in the literature. HbSE disease can be phenotypically similar to sickle B+ thalassemia (HbS/B+thal) and typically presents in adulthood. It may present as a vaso-occlusive crisis (VOC) in the setting of an acute stressor, such as infection, ischemia, toxic metabolic abnormalities, and high altitude [1-5]. We present a case of undiagnosed HbSE disease presenting as a massive splenic infarct in the setting of high-altitude exposure.

## Case presentation

A previously healthy 55-year-old female of South Asian descent, whose medical history was significant only for hypertension, presented to the emergency department with left upper quadrant abdominal pain and hematuria. Her pain initially began four days prior to her presentation while in Switzerland hiking a mountain at approximately 11,000 feet above sea level. During her flight back to the United States, she experienced worsening left abdominal pain and subsequently presented for evaluation. Apart from her recent travel history, social history was non-contributory. On presentation to the emergency department, she was tachycardic to 120 beats/minute, normotensive, and breathing on ambient room air. Physical examination showed a well-nourished, middle-aged female in mild distress from pain. Her abdomen was tender in the left upper quadrant and epigastric region without rebound, rigidity, guarding, or appreciable hepatosplenomegaly. The remainder of the physical examination was unremarkable. Family history was concerning for sickle cell disease among distant members but not first-degree relatives. She denied ever receiving formal genetic testing.

**Figure 1:**
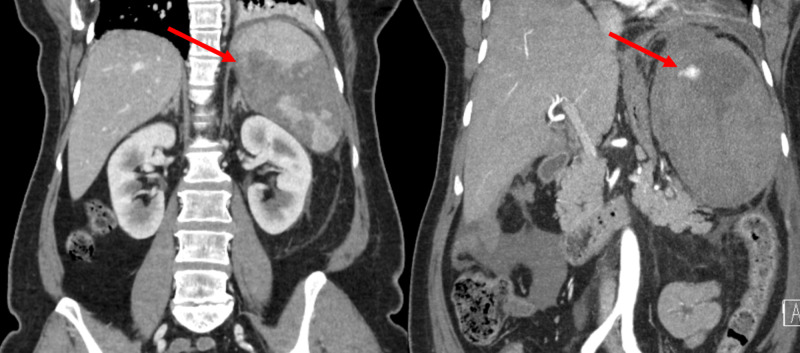
Initial computed tomography (CT) abdominal Imaging Left: Initial CT imaging with the spleen measuring 14.3 cm x 9 cm and an infarct involving > 50% of the parenchyma (red arrow). There is edema surrounding the spleen. Splenic artery and vein are patent. Right: Hospital day 3 contrast-enhanced scan during the arterial phase. Most of the spleen is non-enhancing, compatible with infarction. There is substantial interval worsening of the subcapsular/perisplenic hemorrhage/hematoma formation with the entire process measuring 15 x 10 cm in size now. Active contrast extravasation within the area splenic hemorrhage is demonstrated superiorly (red arrow).

Initial laboratory tests showed a leukocytosis level of 21,900/mm^3^ with a 75.8% neutrophilic predominance, microcytic anemia (hemoglobin 11.7 g/dL, mean corpuscular volume (MCV) 72 fL), and thrombocytopenia (122,000/µl). A blood smear showed 1% - 5% polychromasia cells and 1% - 5% crenated red blood cells (RBCs). A complete metabolic panel showed an elevated anion gap to 15, without elevated lactate, and transaminitis (serum aspartate aminotransferase (AST) 172 u/L, alanine aminotransferase (ALT) 151 u/L, and alkaline phosphatase (ALP) 212 u/L). Urinalysis showed 2+ blood, 2+ bilirubin, and 3+ protein. Workup for hemolytic anemia revealed an undetectable haptoglobin (< 8 mg/dL), elevated lactate dehydrogenase (1,107 u/L), a direct hyperbilirubinemia (2.3 mg/dL), and total bilirubin (5.0 mg/dL). Reticulocyte count was appropriately elevated at 34,000 u/uL with an immature reticulocyte fraction of 31.7%. An initial CT abdomen revealed splenomegaly to 14.3 cm and an infarct involving greater than 50% of the splenic parenchyma (Figure 1). Work-up for hemoglobinopathy in the setting of a suspected vaso-occlusive crisis was initiated. After consultation with hematology specialists, anticoagulation with enoxaparin, 1 mg/kg twice daily, was initiated based on current, albeit mixed data, supporting the use of anticoagulation in sickle cell patient's on a case by case basis.

On hospital day 3, she developed severe distress with worsening of the left upper quadrant pain with radiation to the epigastrium. She was tachycardic to 133 beats/minute and tachypneic to 33 breaths/minute with new hypoxemia requiring supplemental oxygen by 2 L/min nasal cannula. Her abdominal exam was significant for worsened left-sided abdominal and epigastric pain, now with guarding and rebound tenderness. Repeat laboratory work revealed worsening leukocytosis to 31,600/mm^3^ with a neutrophil predominance of 84.2%, an overnight progression of anemia from the initial hemoglobin of 9.3 g/dL to 6.2 g/dL without an obvious bleed, and new thrombocytosis to 436,000/uL. A bedside point of care lactic acid was elevated to 4.0 mmol/L. An emergent CT angiogram was ordered and was concerning for an active hemorrhage of the initial splenic infarct (Figure 1). She was transfused with three units of uncross-matched packed red blood cells and underwent vascular intervention with coil embolization of the involved splenic artery branch. Working diagnoses at this point included a vaso-occlusive event in the setting of an unknown hemoglobinopathy, thromboembolic phenomenon, or undifferentiated hemolytic anemia.

Hemoglobin electrophoresis later revealed HbS 68.6%, HbE 26.3%, HbA 0.0%, HbA2 3.7%, and HbF 1.4%, suggestive of hemoglobin SE disease. The diagnosis was confirmed on capillary electrophoresis (Table 1). She was immunized for functional asplenism and monitored for four days before discharge home.

**Table 1:** Hemoglobin Electrophoresis Percentages for Various Hemoglobin Variant Diseases HbA: hemoglobin A; HbA2: hemoglobin A2; HbAE: heterozygous hemoglobin E disease; HbAS: sickle cell trait; HbC: hemoglobin C; HbE: hemoglobin E; HbE/B thal: heterozygous hemoglobin E and B0/+ thalassemia disease; HbEE: homozygous hemoglobin E disease; HbEF: Bart’s EF disease; HbF: fetal hemoglobin; HbS: hemoglobin S; HbSC: hemoglobin SC disease; HbS/B0thal: heterozygous hemoglobin S and B0 thalassemia disease; HbS/B+ thal: heterozygous hemoglobin S and B+ thalassemia disease; HbSC: hemoglobin SC disease; HbSE: heterozygous hemoglobin S and E disease; HbSS: sickle cell disease

Variant	HbA	HbA2	HbF	HbS	HbE	HbC
Normal	95 - 98%	2 - 3%	< 2%			
HbAS	50 - 60%	< 3.5%	< 2%	35 - 45%		
HbSS		2 - 4%	2 - 15%	80 - 90%		
HbSC		< 3.5%	1 - 8%	45 - 50%		45 - 80%
HbEE		> 3.5%			70 - 90%	
HbAE	40 - 60%	< 3.5%			20 - 40%	
HbEF			10%		80%	
HbS/B0 thal		> 3.5%	2 - 15%	80 - 90%		
HbS/B+ thal	5 - 30%	> 3.5%	2 - 10%	65 - 90%		
HbE/B thal	0 - 20%		30 - 70%		30 - 70%	
HbSE		2 - 4%	< 2%	60%	30%	
Our Case		3.7%	1.4%	68.6%	26.3%	

**Figure 2:**
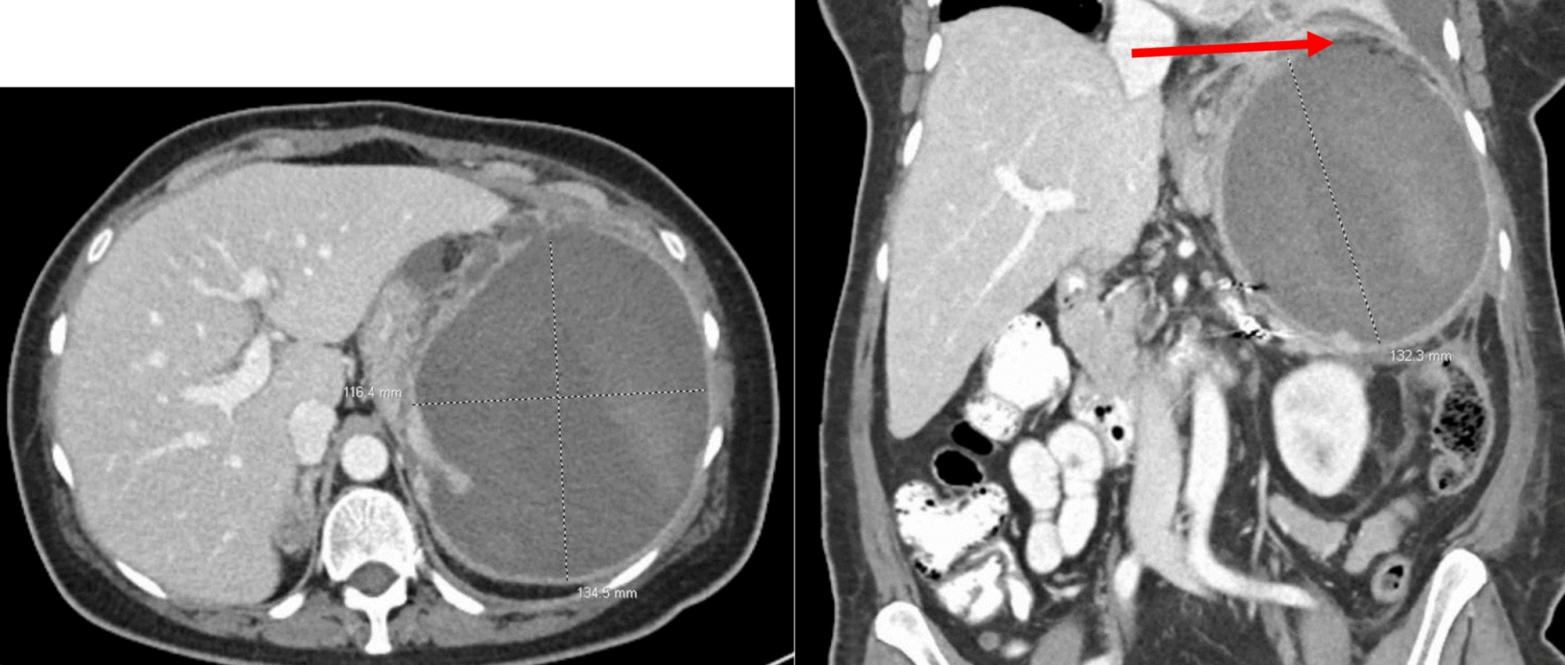
Pre-splenectomy transverse and coronal view of extensive splenic infarction Extensive splenic infarction with a near-complete lack of cortication and necrosis of the spleen. There has been interval enlargement of the spleen, compared to the prior examination, currently measuring 13.5 x 11.6 x 13.2 cm. There are new indistinct margins superiorly indicating partial rupture of the superior splenic capsule (red arrow). Additionally, there is a single nidus of higher attenuation noted superiorly which may represent a small venous hemorrhage versus residual splenic parenchyma, and there is a collection of gas within the necrotic spleen.

Hospital follow-up was routinely performed by various specialists with little change in the patient's clinical status, including follow-up CT imaging. However, seven weeks after discharge, she experienced acute worsening of left upper quadrant abdominal pain and was admitted to the hospital. A CT scan of the abdomen and pelvis showed progression of the splenic infarct with near-complete loss of cortication and necrosis of the spleen, as well as interval enlargement of the spleen (Figure 2). She required an emergent splenectomy with a partial-colectomy at the splenic flexure and left-sided chest tube placement for pleural effusion. Immediately post-surgery, she recovered well with conservative measures of nutrition, mobility, and pain control, and she was discharged home four days later. Follow-up appointments with surgery and hematology suggest optimal wound healing and patient recovery, including appropriate weight gain, increased appetite and functionality, and well-controlled abdominal pain without serious complications. She completed an appropriate course of prophylactic antibiotics in the setting of splenectomy.

## Discussion

Sickle cell trait and disease are the most commonly encountered hemoglobinopathies [6-7]. Among African-American births, the prevalence of sickle cell trait (SCT) is approximately one in 13, while sickle cell disease (SCD) is approximately one in 365 [8]. The substitution of valine for glutamate on the β-globin gene results in a hemoglobin tetramer (HbS) that polymerizes in deoxygenated states, causing erythrocyte sickling and vaso-occlusion [9]. As an evolutionary adaptation against malaria, HbS is most prevalent throughout sub-Saharan Africa and smaller regions of the Mediterranean, the Middle East, and India [6-7]. While almost all SCD patients experience obvious clinical complications of the hemoglobinopathy, most patients with SCT remain asymptomatic [10-11].

Hemoglobin E trait and disease (HbAE and HbEE) are most frequently seen among peoples of East Indian and Southeast Asian descent and are currently the second most common hemoglobinopathy variant worldwide [6-7]. Similarly, it evolved to provide resistance against malaria. Hemoglobin E (HbE) variants occur through the creation of a cryptic splice site on the β-globin gene and substitution of lysine for glutamate, resulting in reduced synthesis of the β-E chain [12]. Clinically, both HbAE and HbEE disease are mostly silent mutations that may result in mild microcytic anemia due to diminished erythrocyte lifespans [12].

**Figure 3:**
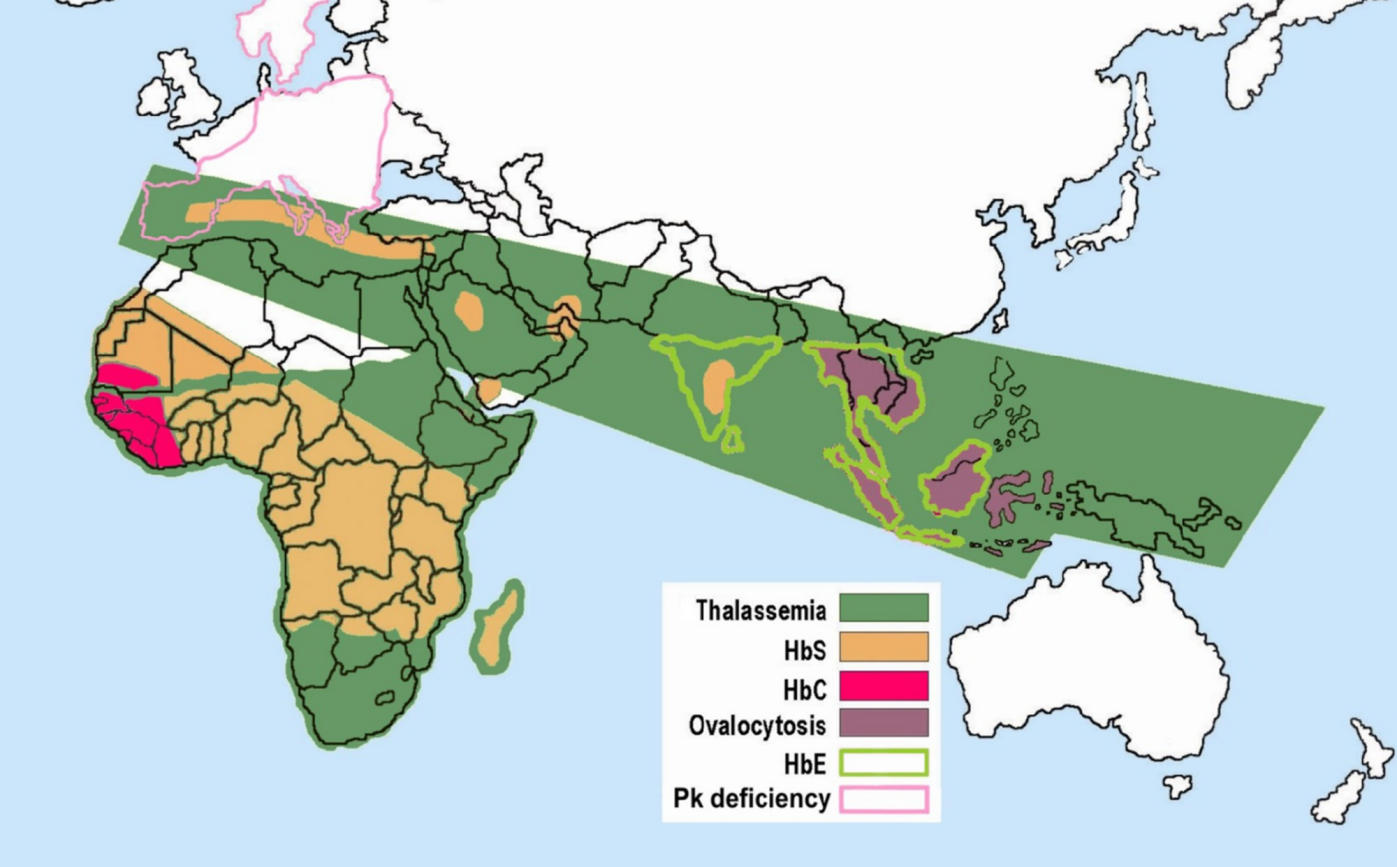
Distribution of hemoglobin abnormalities: Malaria versus sickle-cell trait distributions Copied with permission of the original creator, Armando Moreno Vranich, Wikimedia Commons [14]

Hemoglobin variants have the propensity to form compound heterozygosity. The most frequently encountered variants include hemoglobin sickle/C disease (HbSC) and sickle β+ thalassemia (HbS/B+ thal) [13]. Clinicians encounter compound heterozygous variant hemoglobin SE (HbSE) diseases very rarely, especially in the Western hemisphere. Geographic separation of HbS and HbE makes co-inheritance of the two traits an extremely rare occurrence, and currently, there are fewer than 50 reported cases of HbSE disease (Figure 3) [1, 6-7, 12]. On clinical presentation and initial blood work, hemoglobinopathies and their variants may be indistinguishable and require electrophoresis for an accurate diagnosis. Table 1 presents normal and common variant hemoglobinopathy electrophoretic distributions, reported values for HbSE disease, and the results of the case for comparison. 

It has been postulated that HbSE disease leads to a clinical phenotype similar to that of HbS/B+thal, where symptoms increase in frequency with age [1]. In HbSE, VOCs are thought to be due to an accumulation of subclinical microinfarcts that manifest as a clinical event during adulthood after acute stressors (e.g., metabolic abnormalities, hypoxia, high-altitude travel) [1, 12]. Most reported cases of HbSE disease involve significant sickling-related complications, including acute episodic pain, acute chest syndrome, skeletal infarction, splenic infarction, and splenic sequestration [1]. 

In SCD, splenic sequestration and infarction are common where most patients progress to autosplenectomy by adulthood. Conversely, SCT tends only to present with splenic events in adulthood. To our knowledge, one other case report from 1977 has described high-altitude VOC in the setting of HbSE disease [15]. In that case, a previously healthy 22-year-old male suffered a splenic infarction while aboard a flight and was subsequently found to have HbSE disease. Since there tends to be an increased ratio of HbS: HbE in HbSE disease, it is suspected that vaso-occlusion occurs by a similar mechanism as seen in SCD [16].

Management guidelines for HbSE disease do not exist. Since complications of this disease arise from HbS and not HbE, management should be in accordance with SCD guidelines. While VOC may lead to infarct, such as witnessed in this case, the use of anticoagulation therapy is debated and should be initiated on a case-by-case basis. There are no definitive recommendations regarding antiplatelet or anticoagulant therapy in either sickle cell trait or sickle cell disease patients. Studies have been unable to show an impact on crisis duration, symptom control, or prevention of recurrence [17-18]. In patients with hemoglobinopathy, the most common etiologies of infraction include inherent hypercoagulability and the result of a cardioembolic event. Therefore, as hemoglobinopathy was suspected but not confirmed, anticoagulation was empirically started to prevent additional potential cardioembolic events from occurring [17-18]. Conservative management, fluids, and analgesia are typically adequate for resolution of symptoms as infarcts are due to “sludging” of sickled cells [9, 17]. However, some reports exist of true thrombus formation [19]. In regards to prophylaxis, hydroxyurea is first-line therapy for pain episodes in sickle cell disease; thus, a trial of hydroxyurea may be considered in hemoglobin variant patients with frequent or recurrent episodes. Hydroxyurea leads to an increase in the synthesis of fetal hemoglobin (HbF) and an inhibitory effect of HbF on the polymerization of sickle cell hemoglobin. The hemoglobin shifts which occur as a result of hydroxyurea therapy remain unknown in the HbSE population. Additionally, because HbE is known to be oxidatively unstable in vitro, it may be beneficial to avoid prescribing oxidant drugs in these patients, such as antimalarial agents [1]. The decision to transfuse during a VOC should be approached judiciously based on the clinical picture. Generally, transfusion should be considered since it can be a life-saving measure [20].

## Conclusions

Hemoglobin SE disease is a rare heterozygous hemoglobinopathy resulting from the combination of hemoglobin variants geographically separated by thousands of miles. This variant has a variety of presenting phenotypes, ranging from clinical silence to severe VOC, resulting in infarction. HbSE disease typically remains silent until adulthood and may present as an infarction from a significant stressor. Currently, there are no strict guidelines supporting anticoagulation for the management of VOC in hemoglobinopathies. The decision to begin anticoagulation should be approached from a multifactorial perspective and with the assistance of an anticoagulation expert. Major indications for consideration of anticoagulation in this subgroup of patients include symptomatic venothromboembolus, acute chest syndrome secondary to pulmonary emboli, type 4 pulmonary hypertension, and history of splenectomy. Infarction in HbSE disease should be managed similarly to SCT/SCD, with fluids and analgesia. Anticoagulation poses a high risk for life-threatening hemorrhagic conversion, so the decision to start anticoagulation should not be taken lightly and should be initiated only with the help of experts.
